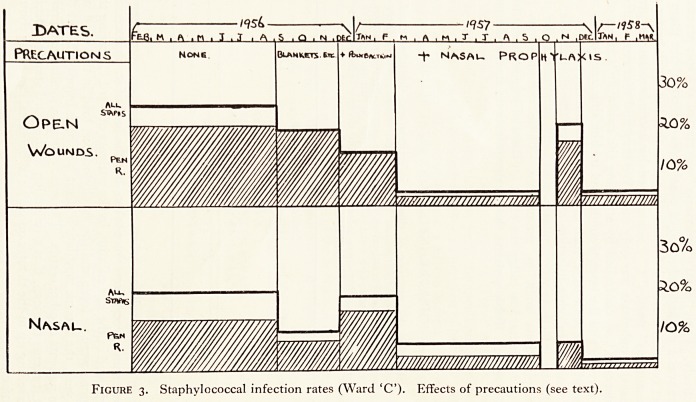# Hospital Cross-Infection
*From an address to the Bristol Medico-Chirurgical Society, on May 21st, 1958.


**Published:** 1958-07

**Authors:** W. A. Gillespie

**Affiliations:** Pathologist, United Bristol Hospitals


### HOSPITAL CROSS-INFECTION*

W. A. GILLESPIE, M.D., F.R.C.P.I., D.P.H.
*Pathologist, United Bristol Hospitals*

It is customary to begin a talk on Hospital Cross-Infection by quoting the well-known words of Florence Nightingale. In 1859 she said that "It may seem a strange principle to enunciate as the very first requirement in a hospital, that it should do the sick no harm".

Her words are still relevant today in spite of all our improvements in asepsis. Hospitals may still be dangerous places, although much less dangerous than in Miss Nightingale's day.

Cross-infection in a hospital does not differ essentially from the cross-infection which goes on all the time in any community such as a household or a school. Microbes, both pathogens and non-pathogens, frequently pass from person to person. Most of this cross-infection is harmless; harm comes, however, when the balance between the microbes and the hosts is upset. In hospitals, the balance is often tilted in favour of the microbes, and it is easy to see why. Firstly, the resistance of the patients to infection may be low, for example very young infants, old people, patients whose tissues are exposed by surgical operations, or whose immunity is lowered by debility. Next, in hospitals, there may be very large numbers of pathogenic microbes, produced by patients who themselves are suffering from open infections; moreover, the patients are close together and frequently handled by a common staff, so that the germs can spread easily. Finally, the use of antibiotics has upset the balance among the microbes themselves, and has favoured the spread and multiplication of strains which have developed resistance to the drugs.

There are several important manifestations of cross-infection in modern hospitals, with which I cannot deal in this paper—for example, gastro-enteritis in infants' wards and nurseries. I want to talk about sepsis in surgical and maternity hospitals. Previously, the group-A haemolytic streptococcus was the most dangerous pyogenic organism. It still is a danger, particularly in obstetrics, plastic surgery, and among rheumatic fever convalescents. But nowadays, most septic cross-infection is caused by microbes which have developed resistance to the antibiotics—the staphylococci, the coliforms, and other gram-negative bacilli. And it is with these that I want to deal.

When considering cross-infection, we should try to answer a number of questions:

(i) How important is it, clinically? How much harm is it doing? For example, is it really a fact, as a Scottish bacteriologist is reported as saying, that maternity hospitals are really very dangerous places to have babies in, because of over-crowding and cross-infection? It is very easy to exaggerate the harm done, and to forget the benefits of hospitals. We need a statistical evaluation of the harm done by cross-infection, in terms of death and ill-health, and delayed wound healing.

(ii) How much are the "hospital" organisms spreading to the community outside, and bringing with them their antibiotic resistance?

(iii) How should the infections be prevented? Is it worth-while trying to do so? Here we find dispute and confusion, because of a serious lack of epidemiological knowledge. Usually, there are several sources of the responsible organism, and many routes by which it may spread. But some of these sources and routes may be far more important than others, and unless we know which, we can use up our energy in trying to introduce a whole lot of remedies, instead of concentrating on the few immediately important things. When faced with sepsis resulting from cross-infection, it is only too easy to make a long list of remedies, all desirable in themselves, but many

* From an address to the Bristol Medico-Chirurgical Society, on May 21st, 1958.

of them costly, or impracticable, or of minor importance in the immediate circumstances. We talk about misuse of antibiotics, carriers among the nursing staff, faulty ventilation, bad wound dressing technique, lack of isolation facilities, dusty floors and blankets, faulty sterilisers, over-crowding, inadequate sluice rooms, toilet facilities, and so on. I do not suggest that these things are not important; but in a particular epidemic, one or two may be much more important than all the others, and should be tackled first. This sort of quantitative information is essential, and can only be obtained by epidemiological study, which may take months or years.

Now I want to illustrate these points by briefly describing some recent investigations in the United Bristol Hospitals. Fuller accounts will be published elsewhere.

### I. URINARY INFECTIONS AFTER PROSTATECTOMY

Some years ago Mr. Ashton Miller suggested that we should investigate this problem, and for three years we have done so. (Gillespie, 1956; Miller *et al.*, 1958).

It is well known that infection is common after bladder operations. But we did not realise just how common it was until we examined daily urine specimens, and found that nearly 75 per cent of patients who came to operation with sterile urine developed heavy infections after prostatectomy. Sometimes the infections cleared up quickly, but in two thirds of the patients, infection lasted for up to three months and in one-third for six months, and in a few cases it lasted for more than a year. The causative bacteria were usually gram-negative bacilli; but staphylococci and Str. faecalis were also common. How important were the infections? Sometimes they are regarded as almost normal or at any rate inevitable complications of prostatectomy. It is true that most cases clear up when the operation site heals, and no great harm is done. But an important minority do suffer serious consequences such as pyelonephritis, endocarditis and septicaemia. In three years we have seen one patient with bacterial endocarditis and nine with septicaemia, all caused by post-operative urinary infection; four of these patients died. The only way to avoid the occasional serious consequences of these urinary infections is to prevent every urinary infection in these patients.

The mechanism of invasion of the blood stream was interesting. The key was the clinical observation that patients sometimes have a rise of temperature, and sometimes a rigor a few hours after removing their indwelling catheters. Mr. Slade and Mr. Linton took blood cultures from a number of patients. From each they took three cultures, the first just before withdrawing the indwelling catheter, the second five minutes later, and the third half an hour later (Slade, 1958). Patients with sterile urine had negative blood cultures; but seven of fourteen patients who had developed infected urine in the few days since their operations, were found to develop a transient bacteraemia five minutes after the catheter was withdrawn. (Table 1). None of these patients came to harm, but there is clearly a risk that the bacteraemia may persist and lead to septicaemia, bacterial shock, or pyelonephritis. A kidney already damaged by prostatic obstruction would be particularly susceptible to pyelonephritis

TABLE I

BLOOD CULTURES 5 MINUTES AFTER REMOVING INDWELLING CATHETERS

Showing that withdrawing the catheter caused a bacteraemia in half the patients when the urine was infected.

| Number of Patients | Urine | Blood Cultures | |
|---|---|---|---|
| | | Pos. | Neg. |
| 15 | Sterile | 0 | 15 |
| 14 | Infected | 7 | 7 |

from blood-stream infection. These blood culture results recall the bacteraemia caused by dilating urethral strictures, demonstrated many years ago by Scott (1929) and Barrington and Wright (1930).

*Prevention.* The interesting findings were that most of the organisms were abnormally resistant to antibiotics and that they did not come from the patient's own bowel. They were cross-infections from other patients. We took many swabs in the wards, and could readily isolate the causative organisms there. We failed to isolate them in cultures from theatre equipment. So we decided to concentrate first on preventing infection in the ward, after operation. We did controlled trials of closed aseptic methods of draining and irrigating the bladder. The old way was to connect the indwelling catheter, placed in the bladder at operation, to a rubber tube which drained into an open pail at the bedside, and if clots caused obstruction, to wash them out with a sterile bladder syringe. Many people as well as ourselves have believed that washing out the bladder in this way may introduce infection. We also found that the open drainage, even without irrigation, may permit infection. It may seem strange that even non-motile bacteria can travel up several feet of tubing, against the flow of urine. We found the explanation in the air bubbles which pass up the tube from time to time, and carry up bacteria.

Our new apparatus was meant to avoid these risks (Figure 1). Urine drains into a closed sterile bottle containing formalin. When clots block the catheter, they are dislodged by sterile saline, allowed to flow in by gravity. If this is not enough, extra pressure or suction is obtained by a Higginson syringe, from which the valves are removed, and which connects the catheter to the drainage bottle. By pinching the tube above or below the bulb, one can get extra pressure or suction and usually dislodge the clots.

For a year we used this entirely closed method on randomly selected patients, while the others had the old open system. The result was a disappointingly small improvement (Table 2). The table shows that the closed cases had a 57 per cent infection rate,

TABLE 2

INFECTION AFTER PROSTATECTOMY

| | Operations | Infections | Infection Rate |
|---|---|---|---|
| Old Methods in Theatre and Wards | 57 | 42 | 74 per cent |
| Closed Drainage and Irrigation . . | 44 | 25 | 57 per cent |
| Same, with new theatre routine . . | 37 | 8 | 22 per cent |

as compared with 74 per cent in the open cases. However, Mr. Linton found that many of the infections were starting within eighteen hours of operation, suggesting that they were introduced at operation, and these theatre infections were hiding much of the good effect of our closed apparatus. Therefore we next directed our attention again to the theatre. As I said before, cultures from cystoscopes etc. had been made from time to time, and had been sterile. But even so, these instruments seemed to be the likeliest vehicles of infection. Some patients on the operating lists already had infected urine, and they were not always taken last. By comparing the properties of bacteria, we found several examples of cross-infection within the lists. Cystoscopes were being disinfected between cases by washing and immersing in oxycyanide, which is a poor disinfectant. Last November, we changed to a mixture of "Hibitane" and oxycyanide for instruments which cannot be boiled. This change brought about an immediate improvement, and reduced the infection rate to 22 per cent.

This investigation is still going on and we are hoping to reduce cross infection further. But we have done enough to realise that organisms have relatively few routes of entry into new patients, and that these routes can readily be controlled. It has paid to direct our main attack on these vehicles and routes, namely the cystoscopes and the drainage apparatus.

FIGURE 1. Apparatus for closed bladder drainage and irrigation after prostatectomy.

### II. STAPHYLOCOCCAL CROSS-INFECTION

Staphylococci have always been important causes of sepsis, but up to fifteen years ago they were overshadowed by the group A streptococci. Since then, the streptococci have remained sensitive to antibiotics, but many staphylococci have become resistant, and hence they are now the chief organisms of sepsis. We are all familiar with penicillin-resistant staphylococci. The curious thing is that although we now see far more of them than formerly, no staphylococcus has become resistant to penicillin. The explanation of this apparent paradox is the fact that even before penicillin was introduced, about 5 per cent of staphylococci were naturally resistant to it because they produced the enzyme penicillinase, which destroys penicillin. These strains have multiplied in hospitals, where penicillin is used, and now amount to over 80 per cent of the staphylococci which we isolate from in-patients and nurses. It is not only the penicillin given to patients which causes the trouble, for Gould (1958) has shown that there are traces of it in the hospital atmosphere, derived from filling syringes, which may have a similar selective effect.

Staphylococci were at first sensitive to the other common antibiotics, but resistant strains have arisen by a process of mutation, and they too have spread and multiplied in hospitals where the drugs are used. But as penicillin is still the most commonly used antibiotic, no staphylococcus can get very far unless it is resistant to penicillin. This is why we find that the staphylococci which are resistant to sulphonamide, streptomycin, tetracycline, and so on, are nearly always resistant to penicillin as well. Clearly, it is the production of penicillinase by staphylococci which is at the root of the problem. If only the chemists could modify the penicillin molecule and make it insusceptible to penicillinase, the staphylococcal problem would be solved.

These considerations rightly make us think about our policy with antibiotics. It is true that antibiotics are sometimes given unnecessarily, and we should be highly critical of such misuse. But to suppose that the problem of the hospital staphylococcus could be solved by being more sensible with antibiotics is, I'm afraid, an over-simplification. It is, of course, particularly important to avoid using the newer antibiotics when possible, so that they may be available to treat the occasional very dangerous infection. We like to keep erythromycin and novobiocin in reserve as far as possible, and to remember, incidentally, that drugs such as "Sigmamycin", "Spiramycin" and "Rovamycin" are relatives of erythromycin and their use may breed staphylococci with a resistance to erythromycin too.

When one investigates staphylococcal cross-infection, one often finds that there are a number of different strains of staphylococcus concerned. Sometimes, however, the trouble is caused by one strain, with exceptional virulence. As Anderson and Williams (1956) pointed out, it is important to decide this point, by phage-typing representative cultures, because the remedy may become obvious; thus if one strain is causing the trouble, it may be possible to identify and treat the carriers and the open lesions from which this organism is spreading, and not expend too much energy in chasing other staphylococci. An outbreak of infection by a Type 80 staphylococcus in the Bristol Children's Hospital illustrates this point (Gillespie and Alder, 1957). It was introduced by children transferred from another hospital in March 1956, and for three months it spread rapidly and caused more infections than all the other staphylococci in the hospital put together. The infections were unusually virulent, and caused the deaths of two children already ill and all non-urgent surgery had to be postponed. Luckily this Type 80 strain was easy to isolate and identify by phage typing, and we were able to tackle it by a campaign of repeatedly swabbing all the noses and open lesions in the hospital. When we found a nasal carrier, we treated him with an antiseptic ointment which usually suppressed his carrier state. Infected patients were barrier-nursed; the organism then disappeared, (Figure 2). It came back again in a similar way, later, and we have had to adopt a set of standing precautions to detect it, and to prevent it from building up its numbers in the hospital.

### STAPHYLOCOCCAL INFECTION IN SURGERY

For two years, Dr. Ayliffe, Mr. Alder, and previously Mr. Wypkema and Mr. Bradbeer have been studying this problem in three surgical wards, (by kind permission of the consultants). The objects were three: to see how much harm the staphylococci did, under normal conditions, in busy wards; to study the sources and modes of spread of these staphylococci, and to see whether the cross-infection could be reduced by adopting various precautions.

We carried out a regular programme of swabbing and of phage-typing the staphylococci which I need not describe. The investigation has had to be carried on for a

FIGURE 2. Type 80 and other Penicillin-resistant Staphylococcal Infections in Children's Hospital.

long time, to allow for the natural fluctuation in staphylococcal cross-infection, depending on the type of surgery and the virulence of different staphylococci which come and go in the wards. It was found that many wounds became colonised by staphylococci with little or no apparent harm, and this colonisation can only be detected bacteriologically; therefore a combination of clinical and bacteriological methods has been necessary.

Staphylococcal wound infections before effective control measures were taken are shown in Table 3. The closed undrained wounds can only be infected at operation.

TABLE 3

STAPHYLOCOCCAL WOUND INFECTION

Showing the incidence of infection when no special precautions were taken.

| | Total | Infected in Theatre | Infected in Wards |
|---|---|---|---|
| Closed Wounds .. | 1196 | 12 (1 per cent) | 0 |
| Open and Drained Wounds .. .. | 397 | 31 (7.8 per cent) | 151 (38 per cent) |
| Total .. | 1593 | 43 (3 per cent) | 151 |

Open and drained wounds can be infected at operation, or later in the wards, because they often have to have dressings changed. Altogether, about 3 per cent of all wounds developed staphylococcal infection in the theatre, and in a good many of these the results were serious, with wound break-down, and there was one death. The ward infections were far more numerous, (nearly 40 per cent of open wounds) but they were less serious. In many of these open wounds, the staphylococci were growing as saprophytes in discharges due to other organisms. Nevertheless, even the open wound infections sometimes caused delayed healing (Clarke, 1957); and they were very important sources from which staphylococci spread to other patients.

In addition to wound infection, staphylococci sometimes caused other complications such as pneumonia and urinary infection. I will deal with them later.

Epidemiological study showed that the most important sources of the staphylococci were open lesions such as wounds, urine and sputum, and nasal carriers among the patients. Many nurses were nasal carriers, also, but they rarely transformed their staphylococci to patients in the wards, although they sometimes did so in the theatre. (Gillespie, 1957).

New strains of antibiotic-resistant staphylococci were sometimes introduced in the noses or lesions of patients transferred from other wards or hospitals. Several different strains spread in the wards from time to time. One or two strains might occupy a ward for weeks or months, going from patient to patient, and then perhaps disappear. These ward epidemic strains all had one feature in common; they were always resistant to several antibiotics. The ease with which they spread was presumably related to the use of several different antibiotics in the wards. These multiple-resistant ward epidemic strains caused nearly all the wound cross-infection in the wards, and nearly all the staphylococcal pneumonia and staphylococcal urinary infections. They even reached the theatre and caused some of the theatre infections. Our evidence suggests that they were carried to the theatre with the patients, perhaps on their blankets. But most of the theatre infections were caused by strains which hardly ever caused ward infections—strains which were sensitive to all drugs, or resistant to penicillin only. These strains probably often came from carriers in the theatre staff, and were to be found in the theatre air. Thus, epidemiological evidence suggests that theatre infections could be reduced by improving the ventilation, by trying to reduce nasal carriage in the theatre staff, and by reducing the staphylococcal loads in the wards.

*Prevention.* We had no idea what preventive measures would prove effective, so we decided to introduce as many changes as possible into one ward, "C", until we got an effect, and then later, to try to decide why. For the first seven months we did nothing except measure cross-infection. Figure 3 shows the staphylococcal cross-infection rates (as percentages) for all the open and drained wounds and the noses. The method of calculating these rates takes account of differences in the duration of exposure to infection (Clarke *et al.*, 1954). A wound or nose which picked up a staphylococcus in a few days would have a higher rate than one which became colonised only after a longer period. Then, in September 1956, we started our precautions, one after another, and kept them going in the hope of getting a cumulative effect. We began with the disinfection of blankets and pillows after use by every patient, using formalin, and by the treatment of nasal carriers whenever we discovered them. Soon afterwards, we started disinfecting crockery, baths and the ward barber's brushes, and we removed communal towels and gave the nurses "Hibitane" hand cream. In December, we started to spray all open wounds with "Polybactrin", which contains neomycin, bacitracin and polymyxin, in order to prevent their becoming colonised by staphylococci and acting as sources of spread to other patients. With all these measures, there was some reduction in cross-infection, but it was not very convincing. In February 1957, we introduced two further measures, with considerable success. One of these measures was "Nasal Prophylaxis"; the application of "neobacrin" ointment twice a day to the noses of *all* patients, from admission, This usually prevented nasal carriage of hospital staphylococci. The other step we took was to improve our method of disinfecting baths, by adding a solution of Hexachlorophene to the bathwater, with each patient. Immediately, the cross-infection rates for noses and wounds fell and stayed down, until October 1957. Then the ward was emptied, because of an outbreak of influenza, and the

FIGURE 3. Staphylococcal infection rates (Ward 'C'). Effects of precautions (see text).

patients were transferred to another ward in which there were many staphylococci. When the patients returned to ward "C", they brought back some of these staphylococci which spread for a few weeks in November 1957 and then the rates fell again to their previous low levels.

In the other two wards, the cross-infection stayed high until June and September 1957, respectively. Then "nasal prophylaxis" was started as a sole precaution, in each ward. There was a marked reduction of nasal cross-infection, but there was a much smaller effect on wound cross-infection. It seems that the much better result in the "all-out precautions" ward ("C") was caused by a combination of precautions, of which the nasal prophylaxis was only one. We may find that cross-infection among surgical patients is best controlled by attacking the two main sources of the staphylococci, the carriers and the open wounds, and by blocking some of the more obvious routes of spread at the same time, such as the blankets and the baths.

But how much does cross-infection matter? From the clinical side, it is the less numerous theatre infections which matter most, and incidentally the other staphylococcal complications such as pneumonia. These complications happen very irregularly, and it is hard to judge the effect on them of improvement in the rates of ward cross-infection. Table 4 shows the theatre infections among the patients of the

TABLE 4

THEATRE INFECTIONS (S. AUREUS)

Showing the number of theatre infections and the number of operations on patients from three surgical wards. Where there was a high rate of ward cross-infection the rate of theatre infection was 2.9 per cent; one ward (C) was then put on full and effective precautions against cross-infection, and the theatre infection rate fell to 0.44 per cent.

| Precautions . .<br>Staph. "Load" in Ward | None<br>High | Some<br>High | All<br>Low |
|---|---|---|---|
| Ward C. . . . . | 8/286 | 2/227 | 2/450 |
| Ward K. . . . . | 17/548 | 7/338 | — |
| Ward S. . . . . | 16/566 | 4/318 | — |
| TOTAL . . . . | 41/1400<br>(2.9 per cent) | 13/883<br>(1.47 per cent) | 2/450<br>(0.44 per cent) |

three wards, in the first column, when no special precautions were taken; in the next column, when some precautions were taken, but the load of staphylococci in the ward was still high; and in the last column, in the patients of ward C, during the year when full precautions were in force and the staphylococcal load in the ward was low. For half of this year, the theatre ventilation plant had been improved, but this had little effect on the patients of other wards. The steady drop in incidence therefore probably means what it seems to mean, that a reduction in the ward staphylococci and in their means of travelling up to the theatre on patients' blankets, can bring about a reduction in theatre infection.

Table 5 shows the important "non-wound" complications for the three wards when their staphylococcal cross-infection was high, and for ward C during the year when the cross-infection was low. Here again, we had a reduced incidence of complications such as pneumonia—not surprising, since the same strains caused the wound and the other cross-infections. (Urinary infections are not shown, since there were other reasons for their reduction).

TABLE 5

STAPHYLOCOCCAL INFECTIONS
(Other than wound and urinary infections)

| | Staphylococcal "Load" in wards | |
|---|---|---|
| | High | Low |
| Number of Operations | 2,043 | 718 |
| Post-operative Chest Infection .. | 12 | 1 |
| Parotitis .. .. | 1 | 0 |
| Entero-colitis .. | 2 | 0 |
| Furunculosis .. | 4 | 0 |
| Total Complications | 19 | 1 |

*Cross-infection in Infant Nurseries*

Another important field of staphylococcal cross-infection is that in the infant nurseries of maternity hospitals. For two years, Mrs. Tozer and Dr. Simpson have been studying the problem. During the first year, the incidence of clinical staphylococcal infection was 107 in 989 babies (just over 10 per cent of babies). About half the infections were of the skin. Fortunately, many of the lesions were small and their effects trivial, probably because there were few of the more virulent staphylococci (e.g. Type 80) in the hospital.

Bacteriological investigation of the healthy babies showed that staphylococcal cross-infection among them was enormously high, as many others have found both in other hospitals and, to a lesser extent, at home.

Table 6 shows that by the second day, most babies had become carriers of Staph. aureus, in nose, umbilicus or groin. The skin of the umbilical area and groin were very often colonised, and often with very large numbers of staphylococci; 64 per cent of the staphylococci were penicillin-resistant.

TABLE 6

STAPH. AUREUS IN SWABS
FROM 2 DAY OLD INFANTS

| | Number of Swabs | Number Positive |
|---|---|---|
| Nose .. .. | 272 | 121 (44 per cent) |
| Umbilicus .. .. | 223 | 130 (58 per cent) |
| Groin .. .. | 268 | 139 (52 per cent) |

Where did the staphylococci come from? Hardly ever from the mothers, sometimes from the nurses' noses, but usually, from the other babies already in the nurseries. There was an enormous self-perpetuating "pool" of staphylococci on the babies, particularly on their peri-umbilical skin, and these were transferred from baby to baby by the handling of the nursery staff, in spite of the strict rules of hand-washing which were in force, as others have shown (Jellard, 1957*a*).

Thus, the babies who became heavily colonised by staphylococci were the main sources of infection of other babies. But their carriage of staphylococci was also a danger to themselves. The babies who did develop clinical sepsis were usually those who had been heavy skin carriers previously, particularly those who were unlucky enough to have their umbilical areas colonised by one of the more virulent strains, such as Type 80 (Tables 7 and 8).

TABLE 7

Showing that infants who had staphylococci in their nose and umbilicus were more likely to develop skin sepsis later.

| Number of Babies | Nose and Umbilical Swabs (Day 2) | Subsequent Skin Sepsis |
|---|---|---|
| 437 | Both Negative | 6 (1.3 per cent) |
| 55 | Both Positive | 11 (20 per cent) |

TABLE 8

PHAGE TYPE OF COLONISING STAPHYLOCOCCUS AND INCIDENCE OF SUBSEQUENT SEPSIS

Infants who were umbilical carriers of Phage Group I staphylococci more often develop sepsis later.

| Phage Group | Umbilical Swabs | Subsequent Skin Sepsis |
|---|---|---|
| I (Includes Type 80) | 29 | 12 (41 per cent) |
| All others . . . . | 61 | 5 (8 per cent) |

*Prevention.* We tried three measures. Two were directed against vehicles of spread, the blankets and the nurses' hands; the third measure was directed against the main source of the staphylococci, the infants' umbilical areas.

Disinfecting the blankets greatly reduced the numbers of organisms in them, but surprisingly, had no detectable effect on the speed with which the babies picked up staphylococci.

A controlled trial of "Hibitane" hand cream by the nurses went on for nearly a year, first in one nursery and then in another. In each nursery, the result was a moderate but quite definite reduction in staphylococcal cross-infection. Table 9 shows the results in one particular nursery ("Floor 2"). When the nurses used the disinfectant

TABLE 9

Showing the effect on staphylococcal carrier rate in infants when nurses used antiseptic hand cream. Swabs taken on second day of life.

| | Nurses' Hands | | |
|---|---|---|---|
| | No Cream | Hibitane Cream | Dummy Cream |
| Infants' Swabs: | | | |
| Nose . . . . | 76/174 (44 per cent) | 64/179 (36 per cent) | 83/158 (53 per cent) |
| Umb. . . . . | 112/172 (65 ,, ,, ) | 61/179 (34 ,, ,, ) | 92/157 (59 ,, ,, ) |
| Groin . . . . | 119/200 (60 ,, ,, ) | 61/179 (34 ,, ,, ) | 80/158 (51 ,, ,, ) |

cream on their hands, there was a fall in cross-infection, and when they used a dummy cream, it rose again. Whether this modest reduction would justify the cost of the cream would depend perhaps on the clinical severity of the infections. But the results are important in demonstrating that handling the babies is how the organisms are spread. As is well known, infants who are roomed with their mothers, and looked after by their mothers, have less infection (Hutchison and Bowman, 1957).

Our third remedy was the most effective. It was directed against the main source of the staphylococci. Jellard (1957*b*) showed that cross-infection could be reduced by applying Triple Dye to the umbilicus. We found that dusting the umbilicus and abdomen with a disinfectant powder, containing Hexachlorophene, had a marked effect on the cross-infection. First we tried it, with strict controls, in the Premature Baby nursery, and the result was very promising. Then we introduced the method in the "Floor 2" nursery, in February 1958. The cord stumps are simply sealed with "Octaflex" and then dusted every time the infant is changed. Table 10 shows that

TABLE 10

UMBILICAL DRESSING

Dusting the umbilicus with hexachlorophene powder reduces the staphylococcal carrier rate in the nose and groin of infants.

| 2nd Day Swabs | Old Method | New (Hexa-chlorophene powder) |
|---|---|---|
| Nose .. .. | 83/158 (52 per cent) | 10/57 (17 per cent) |
| Umbilicus .. .. | 92/157 (58 ,, ,, ) | 9/57 (16 ,, ,, ) |
| Groin .. .. | 80/158 (50 ,, ,, ) | 6/57 (10 ,, ,, ) |

there was a reduction in staphylococcal carriage, to between a quarter and one-fifth of the previous levels, and that nasal carriage has been affected as well as skin carriage. We think that these are very hopeful results. We consider that to reduce staphylococcal infection in maternity hospitals, the most important step is to prevent colonisation of the umbilicus and the abdominal skin. Second in importance, are steps to reduce handling by nurses, by allowing mothers to care for their babies.

---

One of the most important aspects of the staphylococcal problem is the extent of spread of the staphylococci outside hospitals. How much has this happened? Until about 1950, in Bristol, as elsewhere, there had been remarkably little spread of resistant staphylococci into the population as a whole. Since then, there has unfortunately been an unwelcome increase in these strains. Strains from nasal carriers in general practice, many of them kindly collected by Dr. McConnell, show this trend. It is even more marked in strains causing sepsis. One kind of sepsis coming to our Casualty Department, nearly always caused by resistant staphylococci, is the breast abscess. But these abscesses are really hospital infections, for they occur in women who had their babies in maternity hospitals and developed the abscesses after going home (Table 11).

TABLE 11

STAPHYLOCOCCI OUTSIDE HOSPITAL

Showing the proportion of staphylococcal infections due to penicillin-resistant organisms amongst general practice and casualty patients in the last ten years. 30 per cent of boils and septic hands and 80 per cent of breast abscesses are now due to penicillin-resistant organisms.

| | 1949–52 | 1953–56 | 1957–58 |
|---|---|---|---|
| Nasal Carriers .. | 15/357 (4 per cent) | 16/118 (13 per cent) | 24/171 (14 per cent) |
| Boils and Septic Hands | 6/137 (4 ,, ,, ) | — | 65/218 (30 ,, ,, ) |
| Breast Abscess .. | — | — | 28/35 (80 ,, ,, ) |

When we study the phage types of all the penicillin-resistant staphylococci which have caused sepsis amongst the general population we find that many of the infections are caused by a few rather virulent strains. Maternity hospitals are particularly important sources of these unpleasant organisms, and therefore a widespread and vigorous attack on the staphylococcal problem in maternity hospitals is called for.

Staphylococcal infections in hospitals are now a major concern to public health, and the whole community would benefit if the problem were solved.

I wish to thank Messrs. Hough, Hoseason & Co. for a supply of their "ZAC" hexachlorophene baby powder. and Messrs. I.C.I. (Pharmaceuticals) for Hibitane hand cream.

### REFERENCES

Anderson, E. S., Williams, R. E. O. (1956) *J. clin. Path.*, **9**, 94.
Barrington, F. J. F., Wright, H. D. (1930) *J. Path. Bact.*, **33**, 871.
Clarke, S. K. R. (1957) *Brit. J. Surg.*, **44**, 592.
Clarke, S. K. R., Dalgleish, P. G., Parry, E. W., Gillespie, W. A. (1954). *Lancet*, **2**, 211.
Gillespie, W. A. (1956) *Proc. Roy. Soc. Med.*, **49**, 1045.
Gillespie, W. A. (1957) In *Symposium on Hospital Coccal Infections*, Association of Clinical Pathologists, London.
Gillespie, W. A., Alder, V. G. (1957) *Lancet*, **1**, 632.
Gould, J. C. (1958) *Lancet*, **1**, 489.
Hutchison, J. G. P., Bowman, W. D. (1957) *Acta Paediatrica*, **46**, 125.
Jellard, Janet (1957) In *Symposium on Hospital Coccal Infections*, Association of Clinical Pathologists, London.
Jellard, Janet (1957) *Brit. med. J.*, **1**, 925.
Miller, A., Gillespie, W. A., Linton, K. B., Slade, N., Mitchell, J. P. *Lancet* (In the Press).
Scott, W. W. (1929) *J. Urol.* **21**, 527.
Slade, N. (1958) *Proc. Roy. Soc. Med.*, **51**, 331.

## Figures and Tables

**Figure 1. f1:**
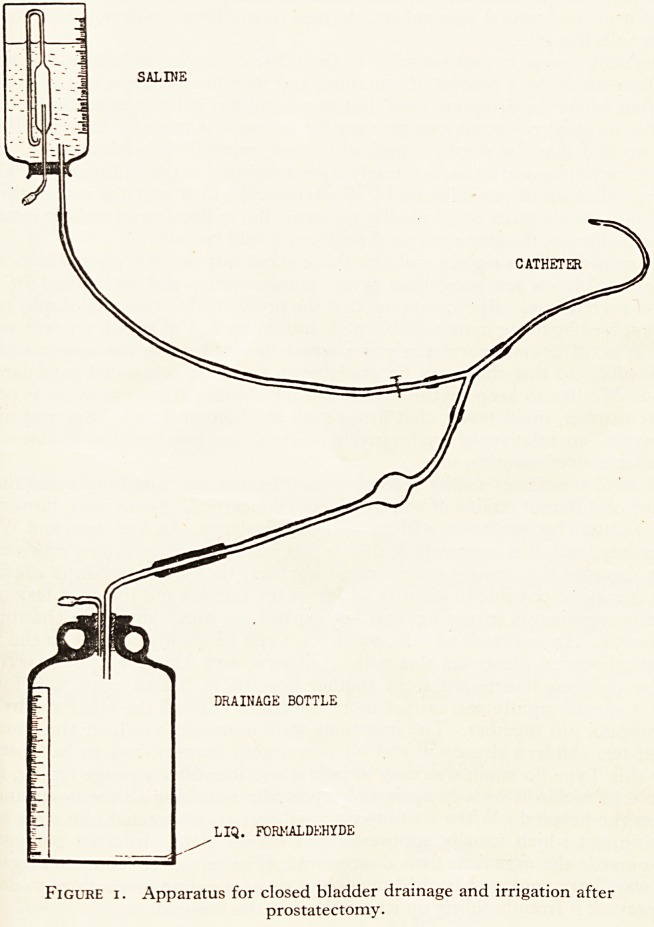


**Figure 2. f2:**
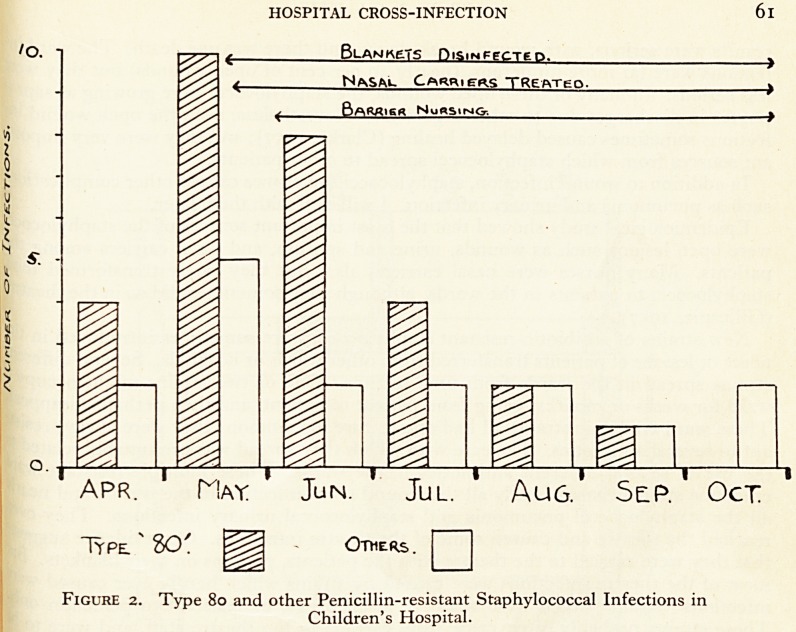


**Figure 3. f3:**